# Guidelines for Reopening a Nation in a SARS-CoV-2 Pandemic: A Path Forward

**DOI:** 10.3390/medicina57050496

**Published:** 2021-05-14

**Authors:** Terrance L. Baker, Jack V. Greiner

**Affiliations:** 1 Johns Hopkins Community Physician, Baltimore, MD 21287, USA; terrancelbakermd@prodigy.net; 2 School of Nursing, University of Maryland, Baltimore, MD 20742, USA; 3 School of Nursing, State University of New York at Stony Brook, Brookhaven, NY 11794, USA; 4 Sollay Medical Center, Sollay Kenyan Foundation, Katani Hospital, Katani, Kenya; 5 Eye Research Institute of Massachusetts Eye & Ear, 20 Staniford St., W239, Boston, MA 02114, USA; 6 Department of Ophthalmology, Harvard Medical School, Boston, MA 02115, USA; 7 Department of Ophthalmology, Tufts University School of Medicine, Boston, MA 02155, USA

**Keywords:** air filtration, COVID-19, guidelines, hand washing, personal protective filtering devices, SARS-CoV-2, social distancing, ventilation

## Abstract

*Background and Objectives*: Action, not fear, is the path forward in the coronavirus infectious disease 2019 (COVID-19) pandemic. Since early 2020, the world’s nations have faced conundrums over severe acute respiratory syndrome corona virus type 2 (SARS-CoV-2) infections resulting in COVID-19 resulting in national closures, and thus, a clear understandable plan that nations can implement is required to reopen. The healthcare benefits of reopening a nation more likely than not exceed the benefits of continued pandemic-related closure. Pandemic-related closures have resulted in countless delayed or avoided urgent care evaluations. Furthermore, routine care of acute and chronic illnesses, including evaluations, diagnoses, and treatments, has also been delayed. Isolation, loss of income, and fear have resulted in mental health conditions or exacerbated existing conditions. The magnitude of untoward ramifications is unknown and may ultimately represent an inestimable degree of danger and morbidity, and even death. The pandemic of SARS-CoV-2 has created an atmosphere of fear of COVID-19 that has directly and indirectly injured the world’s population. Since this has resulted in increasing morbidity and mortality, creating economic chaos, and near systemic collapse of educational systems with no well described plan forward, it is the purpose of this study to provide guidelines that provide a path forward to safely open a nation. Physicians often equipped by their education, training, and experiences across disciplines are uniquely positioned to comprehend, coordinate, and teach other physicians, business owners, and municipal and government leaders from guidelines. As such, physicians may take the lead in a path forward to reopening a nation, including opening businesses, educational facilities, and religious establishments, while minimizing the risk of SARS-CoV-2 infection. *Materials and Methods*: Reviews of the literature among the disciplines of environmental air, sanitation, social interaction, medical testing, vaccination, protection, and disease prevention and safety allowed for the conceptualization and eventual genesis of identifiable interventions which either reduce the viral load in the environment or inactivate the virus from replication. Each of the guidelines was selected based on the principle that it involved the elimination or inactivation of the viral particle. With a reduction in viral load or inactivation of replication, the implementation of these guidelines is expected to allow for reopening a nation with an increased level of safety. *Results*: The guidelines identified, including air exchange (ventilation), air filtration, personal protective filtering devices (masks), hand hygiene, social distancing, screening and testing, vaccines, high-risk patient protection, medical management, and adjunctive therapies, are described and referenced. *Conclusions*: In that the pandemic is primarily a public health issue, the path forward is best coordinated by local, regional, and national physicians. Many physicians with a breadth of experiences are uniquely positioned to coordinate the implementation of these interdisciplinary guidelines. Using these guidelines as a planned, coordinated action, not fear, is a path forward. Nations have a decision to make: closuring versus opening.

## 1. Introduction

The virus responsible for the corona virus infectious disease 2019 (COVID-19) pandemic, severe acute respiratory syndrome corona virus type 2 (SARS-CoV-2), is part of a large family of coronaviruses. The etiology or origin of SARS-CoV-2 remains a mystery, with no clear animal intermediary host confirmed [1,2,3,4]. Coronaviruses are transmitted from person to person primarily by respiratory droplets exhaled by infected individuals [5]. Coronaviruses usually cause mild to moderate upper respiratory tract illnesses such as the “common cold”, with symptoms typically including cough, rhinorrhea, pharyngitis, fever, and fatigue [6,7,8,9]. SARSCoV-2 can cause infections that range from being asymptomatic to having symptoms and signs that are serious enough to manifest in illness that can result in death. As the number of new positive cases of SARS-CoV-2 rises, the death rate due to COVID-19 continues to decline [10] relative to the number of new cases detected. This decline in death rate appears due to improved testing that allows earlier recognition, and thus early treatment and improvements in the medical management of people who develop signs and symptoms of COVID-19. This includes COVID-19 patients severe enough to require hospitalization as well as those treated on an outpatient basis. Early testing, identification, and therapeutic intervention appear to be key to successful treatment outcomes and decreased mortality.

COVID-19 is a highly infectious respiratory disease spread primarily by environmental aerosols that occur as a result of infected people speaking, coughing, sneezing, singing, shouting, exercising or any other vigorous exhalatory effort [11] or even breathing [12]. SARS-CoV-2 spreads like most other respiratory viruses through the inhalation of respiratory droplets. Specific measures are herein recommended to reduce the spread and number of new symptomatic and asymptomatic COVID-19 positive cases. This study combines interdisciplinary discoveries into a set of workable guidelines where such a compilation has heretofore been unavailable. The purpose of this study is to provide a set of guidelines directed to reducing viral environmental load or inactivating the virus so that it cannot replicate. As such, these guidelines may provide a path forward to safely open a nation.

## 2. Methods

A systematic analysis of peer reviewed periodicals and documents published by the WHO, US CDC, FDA, and OSHA was conducted via literature search. This analysis permitted the identification of key strategies that required modification in order to improve the safety of the environment in which society functions that would ultimately inhibit the transmission of the SARS-CoV-2 contagium which can result in COVID-19. With a review of the literature among the fields of environmental air, sanitation, social interaction, medical testing, vaccination, protection, and disease prevention and safety, common themes emerged. These findings more specifically included environmental principles such as ventilation and filtration, medical interventions such as hand washing and masking, and sanitation such as washroom hygiene and fomite disinfection. Recognition of the importance of these fundamental key components led to the arrangement of these elements into a set of guidelines. Each of these principles is part of a common denominator in that each one reduces, eliminates or inactivates the SARS-CoV-2 particle, making the environment safer for patients, staff, and others. The presentation of these interventions required literature searches spanning the fields of medicine, environment and social behavior. Details of each element are provided according to the literature available; however, the guidelines are focused on key principles and are considered to be the minimal necessary components required to safely open a nation. The strategy employed in this study presents guidelines that demonstrate practical methods for improving environmental safety to allow nations to reopen.

## 3. Results

The following specific measures were identified as fundamental principles, each of which either results in the removal or inactivation of SARS-CoV-2.

### 3.1. Air Exchange (Ventilation)

All facilities of all sizes should initiate, on a regularly scheduled basis, air exchange where a high percentage of air within an enclosed environment is exhausted to the outside with the replacement of fresh air. Most facilities currently recirculate their ventilated air, which can contribute to the distribution/spread of the SARS-CoV-2 particle throughout the facility [13,14]. With traditional shared building air exchange systems (ventilation), one infected person in a facility can within minutes contaminate the entire facility [15]. The assumption with air exchange is that the fresh air outdoors is essentially uncontaminated, i.e., free of toxins and pollutants [16]. Examples of air recirculation of SARS-CoV-2 contamination have been reported in hospital rooms and residential apartments [17]. Another example involves cruise ships, in which the recirculation of air on decks and multiple decks results in the contamination of the entire ship from a single infected passenger [18,19]. Examples of air exchange occur in private homes with attic fans capable of exhausting the air from inside the house to the outside. Homes are usually not air-tight and, therefore, replacing the inside air with fresh out of doors air can be accomplished by opening windows. Increasing numbers of new homes are being built energy efficient, and compliance with fresh air ventilation should be part of the energy efficient air management system. Businesses, industries, shopping malls, restaurants, bars, gymnasia, and religious and entertainment establishments can all be modified to circulate fresh air with the goal of completely replacing/exchanging the air within indoor spaces with fresh outdoor air. Public and private vehicles should also adopt air exchange systems that dilute viral particles released in the inside closed compartment(s) of cars, buses, trucks, trains, airplanes, boats and other modalities of transportation. Cabin air exchange by opening windows and/or exhausting internal compartment air to the outside and replacing it with non-contaminated fresh air reduces the risk of infection with SARS-CoV-2 [20]. Regardless of the environment, it is reasonable to suggest that air exchange occurs every hour, although the exchange rate might be tailored to the activity conducted in a space. The Centers for Disease Control recommend room air changes 6–12 times per hour [21]. The purpose of air exchange is to reduce or eliminate the indoor environmental concentration of SARS-CoV-2 particles in order to decrease the viral load and likelihood of infection. The technique of air exchange alone should reduce new infections by removing viral particles from the environment that can readily penetrate the paper or cloth masks being worn by most of the public.

Air exchanges of the indoor environment enhance the efficiency of the cloth or paper filter of the personal protective device by reducing the airborne viral load. By bringing the outdoors, indoors, through air exchange, environmental safety by dilution and reduction in virus particle concentrations, protection from SARS-CoV-2 can be improved.

### 3.2. Air Filtration

Although air exchange is required, the filtering of environmental air is equally important. However, it is likely that the majority of homes and businesses are not meeting the minimum air filtration requirements to filter viruses from the indoor atmosphere [22,23,24,25]. Environmental air filters should meet a Minimum Efficiency Reporting Value (MERV) rating of 13 to 16 to be sufficient to remove SARS-CoV-2 with an average particle size of 0.2 µm [26]. MERV is a rating system developed by the American Society of Heating, Refrigerating and Air Conditioning Engineers to provide a standard method of comparing filters [27]. The higher the MERV rating, the more efficient the filter is at capturing smaller particles. MERV rated filters have documented minimum efficiency reporting values related to a filter’s ability to capture particles between 0.3 and 10 µm. Most standard filters rated less than MERV 13 are only designed to remove dust and large particles from the air [27] and are insufficient for filtering viral particles the size of SARS-CoV-2. MERV 13–16 filters are required to trap SARS-CoV-2 viral particles and virus carriers.

Viral carriers are represented by dust and other particulate matter (PM) in the air that SARS-CoV-2 can bind to and serve as a transportation vehicle. These transportation vehicles can move around the environment until they either transmit infection, fall to the floor, or are trapped by a MERV rated filter or ventilated/exhausted out of doors. A SARS-CoV-2 particle attached to a viral carrier is significantly larger than a SARS-CoV-2 particle alone and can be readily filtered by the MERV 13–16 or an equivalent High Efficiency Particulate Air (HEPA) filter.

MERV rated filters are composed of a tightly woven plastic, glass, or fiberglass mesh, with an open pleated design, whereas HEPA filters are composed of fine fiber glass or plastic threads woven together and compressed to form a tightly closed pleated filter mesh. The MERV and HEPA filters are pleated air filters which work by air flow through the filter fine mesh that traps particles. The pleating of the MERV and HEPA filters increases their surface area, thus improving the chances of trapping viruses and their carriers. The HEPA type air filters remove at least 99.97% of dust, pollen, mold, bacteria and any other airborne particles with a size of 0.3 µm or larger [28,29].

It is important to understand that particles that are equal to or less than 0.3 µm are more difficult to filter. Moreover, these small particles can penetrate even the N95 or N99 personal protective equipment (PPE). For example, up to 5% of these small particles <0.3 µm can penetrate the N95 PPE by definition; up to 1% of these small particles can penetrate the N99 PPE. This penetration of the N95 and N99 PPE is based on a particle size of 0.3 µm [30]. Although the average size of the SARS-CoV-2 particle is 0.2 µm, the N95 or N99 personal protective equipment device (PPFD), due to Brownian movement, can remain effective in filtering particles <0.3 µm.

Brownian movement is the random motion of particles in air including any environmental air, constantly colliding with one another causing redirection. This phenomenon creates random zig zagging motions of virus particles due to the molecular effects of other non-infectious particles in the environmental air [30]. This phenomenon can be applied to both ventilatory and PPE mask filtering systems. These motions make these small light-weight infectious viral particles easier to entrap in the pleats of the MERV or HEPA filters. The non-infectious particles (e.g., dust, lint) in the environment play an important role in the Brownian movement and travel of the viral contaminated droplet aerosols. These non-infectious particles reduce the smaller infectious viral particle’s ability to travel through both filtering systems. This phenomenon would explain the enhancement of the filtration ability of the N95 and N99 masks with particles even <0.3 µm [30]. Brownian movement may not play a significant role in filtration by cloth or paper masks because droplet aerosols may either penetrate or circumvent a loosely applied mask. The concept of the circumvention of droplet aerosols becomes even more notably obvious when employing a face shield alone. As such, the face shield would only provide partial protection because of the even larger open gaps between the shield and the wearer’s facial skin. The importance of a fitted mask such as the N95 and N99 avoids the circumvention of droplet aerosols around the mask edges.

It may be necessary to update air filtration [31] and air exchange [32] systems to allow these viral particles to be removed and inactivated from the indoor environment. MERV-13 is the minimum filter rating recommended to remove viral particles [27]. To be rated MERV-13, a filter needs to remove at least 90% of particles ranging from 3–10 µm in size, 85% of particles ranging from 1–3 µm, and 50% of particles ranging from 0.3–1.0 µm [27]. MERV-16 filters remove 95% of the particles in all three of these ranges. N95 personal protective filter devices (PPFD) (masks) are the equivalent of MERV-16 filters.

Some current heating and cooling (HVAC) systems may not be able to accommodate a MERV-16 filter. Problems may be encountered where the recommended MERV-13 or MERV-16 does not mechanically fit the air handler being used by a current heating and cooling system. Additionally, the MERV-13 filter or higher may add resistance to the airflow in the present system, reducing the total amount of air that the system can move. The result may be lower airflow circulation. Evaluation of the air filtration system by a heating and cooling specialist is recommended.

In addition to the more traditional methods of air filtration, manufacturers are evaluating UVC light applications [33,34] and pulsed blue light applications to inactivate bacterial and viral particles including SARS-CoV-2 [35] passing through exhaust systems. Light application methods would effectively inactivate viral particles before they leave the indoor environment. The use of pulsed blue light may approach 100% inactivation of the virus immediately and, as such, the virus would not remain viable in a filter mesh [33]. Furthermore, the use of pulsed blue light actually cleanses the filter from the virus, thus inactivating viral particles before they are exhausted to the outside air. This would provide additional assurance that the viral particles will be inactivated. It can be important to ensure that the exhausted air from, for example, a restaurant contains only inactivated viral particles, especially in the case of persons passing in the area outside near the exhaust system.

Viral particles can travel in respiratory droplets. A respiratory droplet is a small liquid secretion expelled from the respiratory tract. Respiratory droplets are aerosolized and expelled during exhalation from the mouth or nose. Such aerosolization occurs when a person sneezes, coughs, breathes, sings, and even in the case of rapid deep breathing such as while exercising in a gymnasium, placing the environment at high risk [36]. These aerosolized droplets can be divided into large (>8 µm) droplet particle aerosols (LDPA), medium (2–8 µm) droplet particle aerosols (MDPA), and small (<2 µm) droplet particle aerosols (SDPA).

Large droplets (LDPA) do not spend much time in the air, falling out of the air to the ground quickly, usually within seconds. Droplet falling or grounding is due to droplet weight. Droplet grounding occurs with the vast majority of LDPA within 6 feet from the source person’s mouth or nose [37]. This is the basis for suggesting 6-feet social distancing in order to reduce the exposure and thus spread of a virus. Social distancing reduces exposure to a virus, but it does not eliminate the exposure. With droplet grounding of the LDPA, the large droplets may not be captured by a heating and cooling ventilation system filter in a vent. The LDPA are simply grounded. Although the grounding of the LDPA occurs, it is important to remember that 6-foot social distancing does not ascertain protection from SARS-CoV-2. In the case of both MDPA and SDPA, which also may be larger than viral particles such as SARS-CoV-2 averaging 0.2 µm, these droplets (<8 µm) can rapidly evaporate in the air, leaving behind droplet nuclei that may contain SARS-CoV-2. These droplet nuclei, if small and lightweight enough, can remain suspended in the air for up to 5 h [38].

Once respiratory droplets are exhaled, they move away from the source mouth or nose. While traveling from the source, particles comprising the droplets become less cohesive and disperse [39]. During exhalation, the configuration of the droplets is initially organized in a tightly cohesive pattern. The dispersion of droplets results in LDPA, MDPA, and SDPA based upon droplet size. Although most LDPA fall to the ground within 6 feet, the MDPA and SDPA sized droplets continue to be aerosolized and diluted in the air well beyond 6 feet. This is where social distancing and wearing a cloth or paper mask can be ineffective, since the MDPA and SDPA are able to penetrate the cloth or paper mask with inhalation and exhalation. As such, the cloth or paper mask may not provide protection from inhaling MDPA and SDPA infected particles. Conversely, the cloth or paper mask does not protect others from the inhalation of MDPA or SDPA.

Mechanical air filters such as MERV 13–16 or HEPA can trap suspended SARS-CoV-2 SDPA and MDPA since these filters have an appropriately sized filter mesh. Once the SARS-CoV-2 is trapped by the mechanical air filter, the virus can remain active for hours [39]. This duration of inactivation time is why mechanical air filters should be exchanged with caution as the air filter is potentially contaminated with SARS-CoV-2 and may still be potentially aerosolized and inhaled if the filter is disturbed. For this reason, filter exchange should be performed by experienced staff familiar with the use of full (PPE) [40] and the need to carefully bag soiled and possibly SARS-CoV-2 contaminated filters immediately. It is recommended that most indoor air filters be changed every 90 days to ensure adequate air quality for home or business [41]. Frequently utilized areas of home or business, the presence of pets, or smoking areas may require more frequent filter changes. Rooms of a home or facility may be connected for ventilation and air exchange purposes. Accordingly, all ventilated air coming from a quarantined room should be filtered or rerouted out of doors to avoid contamination and spread [42]. The majority of people reportedly contracting SARS-CoV-2 infections have been found to have visited a restaurant, bar, coffee shop or public restroom in the 2 weeks prior to the onset of their illness [43]. Each of these establishments have a public restroom.

In the case of public restrooms, the restroom door should remain closed when not in use. If windows are present, they should be kept closed as in the open position they can create drafts when the door is opened and if the door is left open. These drafts can disseminate virus particles into the hallway or adjoining space. In the case of public restrooms where doors and windows are closed, the ventilation (exhaust) system should remain active continuously [44]. Since restrooms can be a source of SARS-CoV-2, always wear a personal protective filtering device (mask) when entering and using these restrooms. SARS-CoV-2 particles have been found in urine and feces [44,45]. As such, flushing the toilet can create aerosols of SARS-CoV-2 [46]. It requires multiple additional toilet flushes in order to clear the toilet such that there is no further aerosolization of SARS-CoV-2 [47]. If present, a toilet lid should be in the down position prior to flushing in order to decrease the viral spread via aerosol [48]. This is another reason to wear PPE while in the restroom. The exhaust and filtration systems of the restroom space should complement each other in an effort to complete the task of cleaning indoor environmental air.

The combined goal of air exchange (ventilation) and air filtration is to dilute and remove the infectious viral particles from the environment. All facility, home, and vehicle owners need to have installed appropriately rated MERV or HEPA filter systems with filtering material capable of trapping and removing SARS-CoV-2 particles from the air.

### 3.3. Personal Protective Filtering Devices (Masks)

All individuals need to be encouraged to wear personal protective filtering devices (PPFD) when leaving the safety of their homes and venturing into the community. The PPFD should remain in place continuously until returning to the safety of the home. Environments exist where people believe that they are less vulnerable or safe from becoming infected. These environments include restaurants, bars, churches, and hairdressers where individuals may inappropriately or dangerously doff their PPFD.

Droplet particle aerosols as described above can be divided into three categories according to their size. In the case of large droplets designated as LDPA (>8 µm), cloth masks comprised of multiple layers serve as barriers to inhalation and exhalation. These multilayered masks may stop the movement of LDPA originating from inside or outside the mask.

Medium droplet aerosols (MDPA) measuring 2–8 µm or small droplet particle aerosols (SDPA) measuring <2 µm can be aerosolized for hours since they are lightweight and can travel >30 m [49] from the source (mouth or nose). MDPA and SPDA can attach to carrier particles such as particulate matter (PM) with which they can stay airborne for hours, and that is why ventilation, filtering, and distancing among people are important.

Filtering is important as it relates to the three droplet sizes. All three particle sizes, LDPA, MDPA, and SDPA, can be expressed by an infected patient without a mask. LDPA can fragment into MDPA and SDPA that disperse into the environment [50]. Although LDPA are grounded within 6 feet, MDPA and SDPA require filtration removal. The smaller MDPA and SDPA aerosols can only be removed using MERV 13–16 or HEPA filters. The MDPA and SDPA, once aerosolized, can form droplet nuclei. The droplet nucleus is the core that contains the virus particle(s) formed when the surrounding moisture barrier evaporates from the MDPA and SDPA.

It is important to understand the danger of different types of exhalation. Each breath of a source infected person can result in the exhalation of 200–300 infected droplet nuclei [51]. Exhalation occurring while talking for a period of 5 min will contain approximately 3000 droplet nuclei [11]. Thus, during normal exhalations with breathing or speaking, droplet nuclei are lightweight enough that they are able to hang suspended in the air. Such suspension provides the opportunity for aerosol environmental contamination and spread to uninfected individuals. It is of further interest that a typical cough expels almost 3000 droplets at a speed of 50 miles per hour [11]. A typical sneeze travels up to 100 miles per hour, creating approximately 100,000 droplets [11]. Exhalations during a cough or sneeze allow droplet nuclei suspended in the air to travel at high speeds and well beyond 6 feet.

The outdoor environment can become contaminated with viral containing saliva droplets numbering 200–300 with a normal breath exhalation. Subsequently, these saliva viral droplets can remain suspended in the air for hours after an infected individual has filled the air with hundreds of thousands of viral particles after coughing, sneezing, and singing [52]. This explains the outbreaks, occurring, for example, at the beach, where no masks might be worn. The significant rise in people becoming infected with COVID-19 at a beach [11] may occur with only a few infected people in the area. This infection occurs after walking through air space where people have contaminated the air with droplet nuclei containing viral particles by coughing, sneezing, and simply exhaling from breathing. Cloth or paper PPFD do not prevent the transmission of SARS-CoV-2 [53]. Since a cloth mask can allow aerosol transmission of MDPA and SDPA containing SARS-CoV-2, infected individuals can infect members of the community. This concept is important even for a person walking along a public thoroughfare. An infected person may create a trail and consequently a vacuum containing SARS-CoV-2 active particles. This becomes especially important considering an infected person coughing, sneezing or simply exhaling can create a trail of suspended droplet nuclei containing active viral particles. An uninfected person can subsequently walk through an airspace containing a cloud of MPDA and SPDA, inhaling the suspended droplet nuclei. This explains why one can contract COVID-19 by simply walking on a public sidewalk and why masks should be worn continuously when leaving the home until returning.

Caution is required when approaching a closed entrance door to a closed space such as a room occupied by a quarantined patient(s). When opening the door to enter the room, air currents move from inside the room to outside the room, potentially spreading aerosolized viral particles. Consider that the infected occupant of the room is likely to be unmasked and exhaling with normal breathing, coughing, or sneezing. It is assumed that the LDPA from the infected occupant are grounded or settle on fomites within a 6-feet radius. The exhalations flood the room with countless suspended MDPA and SDPA aerosolized viral particles. Depending on the velocity of opening the room door, except in a room conditioned with negative pressure, air currents will travel from the quarantined room to the environment outside the room. Of concern is that these air currents contain aerosolized viral particles suspended most likely in MDPA and SDPA that can spread from the quarantined room to other parts of a home or facility. This phenomenon of viral spreading can be mitigated with the use of zippered impermeable plastic entries installed in doorways [54]. In addition to air currents, differences in room temperature and humidity also create currents when doors to quarantined areas are opened [20]. Considering the above, wearing masks is required when opening doors to a quarantined room in order to avoid encountering a cloud of contaminated MDPA and SDPA.

Around 40–45% of people infected with SARS-CoV-2 may never have symptoms but can transmit the virus [55]. Viral spread from people without symptoms may account for >50% of all new infections [56]. Masks (PPFD) should be worn continuously when leaving the home and venturing into the community. Masks should remain in place until returning home and should be removed using appropriate techniques as reviewed previously [57] to avoid contamination of the wearer, the home, and family members. Masks should be removed prior to entering the home.

Persons in close proximity wearing a cloth mask can inhale the MDPA and SDPA containing SARS-CoV-2 particles of infected individuals [53]. Most cloth masks and the blue or white surgical paper masks allow the passage of SARS-CoV-2 particles via MDPA and SDPA in and out of the respiratory system as these type of masks have inadequate filtering capability for SARS-CoV-2 particles [58]. The N95–N99 masks filter SARS-CoV-2 particles and reduce transmission and reception of all sizes of particles. As such, a cloth or to a lesser degree a paper mask cannot be expected to serve as a barrier for SARS-CoV-2. However, a cloth or paper mask does serve to reduce the LDPA and to some degree MDPA and SDPA, providing incomplete protection against SARS-CoV-2. These masks are insufficient to filter the MPDA and SPDA created when someone is speaking and/or in close proximity to others in a house, at an office, or at an event. These aerosols originate from and are exhaled from the mouth and nose of the infected person. MDPA and more readily SDPA particles may move through cloth PPFD of an infected source person and thus may be able to initiate an infection [59]. The reason for this ability of the SARS-CoV-2 virus particle to penetrate the cloth or paper PPFD has to do with its small size averaging 0.2 µm [60]. N95 and N99 masks are examples of PPFD that have the ability to filter SDPA. Although some of these SDPA can also be trapped or filtered by cloth or paper PPFD and provide some level of partial protection from SDPA, such protection is more than likely inadequate. By trapping LDPA, the cloth or paper PPFD can facilitate the entrapment of some of the SDPA. This is because the SDPA stick to or aggregate with the LDPA. The result of this entrapment is that it decreases the viral load expelled into the environment by the infected person.

Preventing LDPA production is important in reducing the overall number of particles in the air; however, it does not stop the invisible mist of MDPA or SDPA containing fine aerosol clouds of SARS-CoV-2 particles in the air. These droplet particles may remain in the air for hours after an infected person has left the area [61]. Cloth or paper PPFD are part of a comprehensive plan for the path forward; however, their main role is to protect surrounding individuals from being exposed to the LDPA which the cloth or paper PPFD can contain. Wearing a cloth or paper mask can only be expected to reduce LDPA viral load in the surrounding environment, though it may not prevent the SDPA and MDPA containing SARS-CoV-2 being spread into the environment. Therefore, it should be understood that wearing a cloth or paper mask does not eliminate the need for social distancing. Accordingly, the N95, KN95, or N99, which filter 95% or 99%, respectively, are preferred for the best control of the spread of SARS-CoV-2. Everyone should wear a face covering in order to reduce the spread of SARS-CoV-2. When making adjustments to the PPFD (masks), the head straps or ear loops behind the ears or behind the head should be used [57]. If the mask material is touched with the hands, hand hygiene should occur at the first opportunity [46].

Multiple factors affect mask efficiency, including the quality and composition of the fabric, respiratory rate, relative humidity behind the mask’s inner surface, the temperature of the environment in the retro mask space, and the time-duration of mask wearing, which is related to loading microorganisms into the mask [30]. These factors can affect filtering performance and thus the ability to protect the wearer from microorganisms including SARS-CoV-2. These factors can accelerate viral penetration of the mask. A clean mask daily is an important part of reopening a nation, accomplished either by replacement with a new mask or a recycled mask when this is not possible. As reviewed previously, where appropriate, methods of recycling include exposure to ultraviolet radiation, machine cleaning with hot soapy water, dry cleaning, and exposure to sunshine [57].

### 3.4. Hand Hygiene

Early in the SARS-CoV-2 pandemic, it was believed that SARS-CoV-2 was primarily transmitted by contact with fomites. It has been assumed, but not documented, that fomites could transmit the SARS-CoV-2 particle to the hands of an unsuspecting person who would then touch their face and nose with hands contaminated with viral particles [62]. This may be important since it is reported that patients touch their face 20–30 times a day unconsciously [63]. With facial-touching, SARS-CoV-2 can be in close proximity to the five body openings of the face (eyes, nares, and mouth) [46], which can serve as portals of entry for a virus. Hand hygiene reduces the potential for infection from touching the face with hands contaminated with the SARS-CoV-2 particles [64]. It is possible that hand hygiene will prevent infection spread from handshakes, hugging, and sharing of fomites [65]. Family, social, and business gatherings with people who do not live together can increase the chances of contracting or spreading SARS-CoV-2 [5]. It appears that the vast majority of people in these types of gatherings appear to become infected by aerosolization with LDPA, MDPA and SDPA.

The best form of hand hygiene involves washing the hands with soap and water for 20 s [57]. Hand sanitizers consisting of 60–90% alcohol-based preparations offer the advantage of being readily available and thus time effective. However, alcohol-based preparations also cause concerns about hand health with the development of contact dermatitis and other alcohol-related effects on skin such as dehydration [66]. After sanitizing the hands with either soap or alcohol-based hand sanitizer, the most effective way to dry the hands is to use individual paper towels [67]. Family, social, and business gatherings should observe mask usage and hand sanitization to limit the spread of infection.

Inadvertent mask touching should always be immediately addressed by performing hand hygiene, since hand contact with the mask can result in the spread of active viral particles. This occurs because after touching the mask, the hand subsequently can touch the face, neck, and clothing garments, spreading active virus particles. As this pattern occurs, there can be eventual contamination of the entire body. Furthermore, the air currents induced by such hand movements from the mask down the body assist in the spread of viral particles. Moreover, with viral particles on the hands and whatever is touched by the hand there is potential viral transmission.

### 3.5. Social Distancing

A comprehensive plan for managing a virus that is aerosolized should include social distancing. SARS-CoV-2 aerosols can remain in the air and circulate for hours [68] after an infected person has left the area. The aerosolized virus contained in droplet nuclei (MDPA and SDPA) can reportedly travel freely up to 16–20 feet based on airflow currents around the infected space [69]. For example, an entire restaurant can be contaminated by one person dining indoors without proper air filtration and without air exchange. By the end of one hour, the entire restaurant and virtually every part of the restaurant from the cooking area, food preparation, restrooms, air ventilators, air filters, air ducts, surrounding tables, and the floor are all contaminated with coronavirus particles [15]. Accordingly, although social distancing is important, it is not a panacea in and of itself, it is simply a part of a comprehensive plan of keeping patients at risk, safe. Reportedly 80% of all newly diagnosed SARS-CoV-2 patients have been found to have been in a restaurant setting within 7 days of being diagnosed [70]. The current recommended social distancing is 6 feet [71]. This distance is only based upon the fact that most LDPA exhaled by a SARS-CoV-2 infected person without a mask will fall to the ground within 6 feet [71]. Recommendations for determining social distances should include accounting for multiple parameters such as viral load, ventilation and filtering, type of activity, indoor vs. outdoor environment, and masking.

### 3.6. Screening and Testing

Early detection of SARS-CoV-2 is important for identification, quantification, and the implementation of algorithms reducing the spread of the virus. It is important to recognize that the number of respiratory infections may increase seasonally, whether from non-SARS-CoV-2 coronavirus infections, rhinovirus infections and infections with influenza virus A and B, not to mention symptoms of seasonal allergies and asthma type symptoms. Many COVID positive symptomatic individuals believe they are suffering from the “common cold”. As such, these individuals are not screen tested for SARS-CoV-2. Therefore, these individuals may unknowingly act as SARS-CoV-2 spreaders in their homes, places of business and communities in general. Thus, it is likely that most SARS-CoV-2 is being spread by an unknowing carrier with or without the onset of mild to moderate respiratory symptoms including coughing, sneezing, rhinorrhea, pharyngitis, and even low-grade fever. These individuals continue to be active in the community. With only mild to moderate symptoms, many of these patients may not seek testing for COVID-19, resulting in the report of a falsely low number of cases [6,72]. Symptomatic COVID-19 carriers are simply self-treating with over-the-counter medications and improving. These patients with symptoms [72,73] may continue to work, facilitating the spreading of SARS-CoV-2 throughout the workplace and community.

Screening and early testing will allow early quarantine of infected individuals and early protection of the most vulnerable populations. Although rapid antigen tests for COVID-19 are available, providing results in minutes, the cost of these tests is generally high and thus unaffordable. The development of rapid tests that are affordable and allow prompt results, purchased and then performed in the home, would be ideal. Early positive results would require quarantine until confirmed by a physician administered test. Such a development with adequate availability should have a profound influence on limiting the spread of SARS-CoV-2.

The most vulnerable population and largest number of individuals who have died from SARS-CoV-2 include people over 50 years of age [74]. Nearly half of all individuals who have died from the coronavirus to date have been nursing home residents and/or nursing home staff [75,76]. Due to the high rate of infection and death reported in nursing homes, it is recommended that COVID-19 testing of both residents and staff be performed every 3 days [77]. COVID-19 positive residents and staff should be immediately quarantined.

One study reports that individuals who experience COVID-19 have a 99.3% chance of complete recovery [78]. Less than 1% of the infected population requires hospitalization and/or further treatment [79], and the vast majority of hospitalized patients survive. As such, only a small percentage of the population progresses in their illness, resulting in death. Although a portion of infected individuals maintain some morbid sequelae, most of the population that becomes infected with SARS-CoV-2 recovers completely without any complications or sequalae [79]. Though there is limited information on late sequalae of COVID-19, reports of persistent symptoms in persons who recovered from acute COVID-19 illness have emerged [80]. These include fatigue, dyspnea, cough, arthralgia and chest pain [68]. Most patients who die from complications of SARS-CoV-2 have pre-existing serious underlying comorbidities [81], and the protection of these high-risk vulnerable individuals until a vaccination is available is important. Early detection of individuals infected with SARS-CoV-2 allows the opportunity to protect vulnerable citizens from exposure to persons carrying the disease.

Early detection of individuals infected with SARS-CoV-2 can be accomplished by monitoring the oxygen saturation of hemoglobin (O_2_ Sat) [82]. Frequently, patients infected with SARS-CoV-2 will not notice abnormalities of the respiratory rate; however, the O_2_ Sat will decrease to less than 93%. O_2_ Sat testing can be monitored by nursing home staff less expensively than COVID-19 testing. Normal values for O_2_ Sat in room (ambient) air range from 93–100%. Abnormal O_2_ Sat in an otherwise healthy person is less than 93%. O_2_ Sat can be measured using an oximeter not only by the nursing home/facility staff but by primary care physicians, urgent care centers, emergency departments, and business establishments. Online apps are available to convert most smartphones into O_2_ monitoring devices and thus such measurements can be performed by anyone. Pharmacies and on-line vendors also provide affordable and readily available oximeter devices for the measurement of O_2_ Sat. The screening of patients for SARS-CoV-2 and measurement of O_2_ Sat levels can be utilized as part of early recognition of underlying COVID-19 disease and disease progression.

### 3.7. Vaccines

Vaccination is an important part of viral eradication. There are many examples of using vaccination to achieve herd immunity and to reduce the possibility of viral spread [83,84]. Herd immunity in part will be achieved by protecting the world’s populations from SARS-CoV-2 before they become exposed and infected. Accordingly, the world’s population must have a population immunity through vaccination and or immunity created through infection with SARS-CoV-2 to protect at least 67% of the population or more to limit new infections [83,85].

Herd immunity, also known as population immunity, is a concept used to describe protection from a certain virus if a threshold of immunity from that disease is reached. The current thought is that 70% of a population must either be vaccinated or develop natural infection in order to stop the spread of SARS-CoV-2 [83]. However, the effectiveness of viral vaccines is not a given [86].

Coronaviruses are elusive in that these viruses mutate frequently [87], and thus developing an effective long-lasting vaccine can be difficult. For example, the virus that is currently causing the majority of COVID-19 is not the same strain as what first emerged from China [88]. SARS-CoV-2 has mutated (D614G) in a way that makes it more contagious to humans and is now the predominate species around the world [88]. Additional mutations (285) have been identified [89]. Mutations in South Africa, the United Kingdom, Brazil, and the United States of America have been identified. The purpose of these mutations is unknown, although it is presumed to be important to the survival of the virus [90,91,92].

Vaccines against coronavirus type viruses being developed and deployed may only result in short-term, i.e., 3–6-month immunity [93], and thus are not a panacea for success but are expected to curb morbidity and mortality. It is recommended that a vaccine not be administered in patients that have been infected with SARS-CoV-2 within the last 6 months because of the existence of natural immunity. Consider, however, it has been reported that the duration of natural immunity may be limited [94]. Although usually mild COVID-19 disease can be observed in children, the vaccination of children against COVID-19 is being studied and outcomes are pending. The vaccination of front-line workers should be implemented first, followed by vulnerable individuals and then the general public. COVID-19 vaccination provides a level of protection against developing severe COVID-19 disease and death from SARS-CoV-2 infection. Side effects after vaccination [95,96] are typical signs that the body is building protection. Although world-wide vaccination is underway, data concerning possible side effects and reactions are currently at the data collection stage.

### 3.8. High-Risk Patient Protection

The protection of high-risk patients is a key concept which is perhaps easier to discuss than in reality to achieve. Of the COVID-19 related deaths to date, a large number of these deaths have occurred in patients over 50 years of age with comorbidities. Many of these deaths occurred in patients with multiple comorbidities residing in nursing homes, assisted living, and rehabilitation facilities. Medical conditions such as obesity, coronary artery disease, hypertension, cancer, immune suppression, and diabetes mellites have been related to an increased rate of becoming infected with increased complications including death [81]. Over 40% of all U.S. SARS-CoV-2 deaths involve long-term nursing home or assisted living residents, and rehabilitation patients including staff members [97]. Although all nursing home residents and rehabilitation patients including staff may be vaccinated, individuals identified as vulnerable and, as such, at risk need to be quarantined and isolated to the extent possible.

Another consideration for the protection of high-risk individuals is aging. For example, many members of a workforce are designated essential workers [98] currently 50–70 years of age [99]. These workers in this age group are vulnerable to the SARS-CoV-2 disease process. Members of this age group are skilled essential workforce required to keep a nation open and productive. For example, in health care alone, the nursing staff may include a high percentage of individuals in this age group [100,101]. Quarantining all individuals 50–70 years old with risk factors of age and comorbidities is impractical. However, to the extent possible, this high-risk population does need to be identified by all primary healthcare providers, all facilities and all families so that these individuals can take precautions to shield or limit their exposure to SARS-CoV-2. This can also be accomplished by providing alternative occupational opportunities in environments with less possible exposure to infected individuals.

Similar caution should also be used by family members visiting relatives who are vulnerable based on age or underlying medical conditions. It is especially important during these visitations to wear masks and social distance. This also includes family gatherings where two or more households come together for celebration or visitation where facial masks and social distancing remain important. As discussed above, it is important to avoid a false sense of security or comfort and not use facial masks or social distancing precautions such as when being in certain more comfortable environments, for example, homes, restaurants, and in places of business.

High-risk patients residing in nursing homes as long-term care residents, assisted living residents, and rehabilitation patients should be protected by facility staff. Facility staff should be adequately trained and equipped with PPE [57] and have access to rapid SARS-CoV-2 testing and the necessary antiviral education to keep residents and the facility staff safe.

### 3.9. Medical Management

Medical management includes early recognition, adequate hydration, monitoring oxygen saturation daily [102], additional supplemental oxygen as needed, and, additionally, therapeutic intervention. Successful therapeutic intervention in the case of potentially fatal viruses has historically included a cocktail approach. The cocktail approach of anti-viral therapeutics (medications) regarding SARS-CoV-2 is the subject of ongoing investigations [103]. The mechanism of action of a cocktail is that combinations of antiviral medicines can be employed for the purpose of interrupting the virus’ life cycle at multiple locations. These location sites in a typical virus include the site of attachment to the host cell receptor, the completion of cellular incorporation (cytoinclusion), uncoding the genome, nucleic acid (RNA/DNA) replication, and the release of replicated viral particles. Interruption at these location sites causing dysfunction can render the viral life cycle ineffective, thereby stopping the replication of the virus and ultimately the disease process. Historically, a minimum of three medications administered simultaneously have been effective for the successful cocktail treatment of cancer [104]. Similarly, when treating HIV, a cocktail of at least three medications has proven to disrupt the viral life cycle and to prevent the disease from progressing [105].

Although vaccines and the use of multiple antibody cocktails [106] are proposed for the mediation of the SARS-CoV-2 pandemic, the possible medical management of COVID-19 employing cocktails of repurposed antiviral medicants should not be overlooked [107]. Drug repurposing is the repositioning of an approved drug for the treatment of a different disease or medical condition than that for which it was originally developed. This drug repurposing and strategy of a cocktail approach has been used successfully for the treatment of both cancer [108] and HIV diseases [109]. The proposed cocktail approach has been reported retrospectively in the treatment of COVID-19 patients [110]. Using repurposed medicants comprising an antiviral cocktail may have utility for the treatment of SARS-CoV-2 and even long-term utility for future pandemics as each medication can disable one or more components of the viral life-cycle. Physicians’ abilities to treat COVID-19 have dramatically improved since this virus first appeared [111]. This is due to a better understanding of early diagnosis, conservative hydration, ventilator management, endovascular complications and the prevention of cytokine storm, resulting in a reduction in the morbidity and mortality of infected individuals.

### 3.10. Adjunctive Therapies

Potential patients and SARS-CoV-2 positive individuals at risk for developing COVID-19 should consider using adjunctive medications [112] known to be important in promoting multiple healthy immune functions. Medications include vitamin D, vitamin C, zinc, quercetin, aspirin, and N-acetylcysteine, all of which act as cofactors in immune functions. Early use or treatment with these medications may reduce the need for hospitalization and the severity of the disease.

- **Vitamin D.** Vitamin D (cholecalciferol) exists in two forms, D_2_ and D_3_. D_3_ is the natural form of vitamin D synthesized by the skin from sunlight. In a society where sunlight exposure is limited, the ingestion of exogenous vitamin D is required. D_3_ is the preferred form of vitamin D since it is nearly twice as effective as vitamin D_2_ for elevating blood serum levels. Vitamin D is a fat-soluble vitamin that plays multiple important roles in the immune system, which modulates the body’s reaction to infection. It enhances the function of immune cells including T cells. For example, it enhances the abilities of monocyte derived macrophages to mature, to produce macrophage specific antigens and to secrete H_2_O_2_, both of which have significant antimicrobial function [113]. Vitamin D inhibits macrophage release of proinflammatory cytokines. Cytokines are proteins that are an integral part of the immune system. They have anti-inflammatory effects that play an important role in protecting against inflammation. However, uncontrolled release of cytokines can cause tissue damage and can lead to cytokine storm. A cytokine storm refers to the uncontrolled release of proinflammatory cytokines that can take place in response to SARS-CoV-2 infection. The excessive release of cytokines leads to severe tissue damage and enhances SARS-CoV-2 progression and severity. Cytokine storm is a major cause of organ failure and advanced respiratory distress syndrome (ARDS) as well as an important factor in COVID-19 progression. Vitamin D deficiency is associated with morbidity and mortality in COVID-19. Vitamin D supplementation to the population at risk for COVID-19 reduces the chance of developing severe disease after infection with SARS-CoV-2 [114]. Vitamin D deficiency is associated with patients with more severe COVID-19, whereas patients with high levels of vitamin D were associated with less severe COVID-19 [114]. The recommended daily allowance (RDA) from 1–70 yrs of age is 600 IU po daily and 70–100 years of age is 800 IU po daily. The prevention of community acquired pneumonia and COVID-19 has been observed to require 2000–5000 IU po daily [115].
- **Vitamin C.** Vitamin C (ascorbic acid) has potent anti-inflammatory and antioxidant effects. A viral infection causes increased oxidative stress manifested as cellular damage. Supplemental vitamin C may prevent coronavirus infections, shorten the disease course, and lessen complications of the disease including the progression to cytokine storm [97]. These mechanisms have been reported in COVID-19 [116,117,118]. Vitamin C is a safe and effective therapy and may dramatically reduce the need for high doses of corticosteroids and antiviral medications as well as being used prophylactically [116].

The RDA is 65–90 mg po daily with an upper limit of 2000 mg po daily. Notably, the recommended dose for prophylaxis against SARS-COV-2 is 1000 mg po q8 hr totaling 3 gm po daily [116]. These elevated doses can cause complications such as nausea, vomiting diarrhea, and pruritis and may require dose reduction. Supervision by a physician is recommended.

- **Zinc.** Zinc is important in the development and function of the immune system. Zinc has been studied for the treatment of viral respiratory infections and is a natural protection against SARS-CoV-2 severity [119,120]. Zinc prevents the formation of platelet aggregates and thus endovascular complications; improves tracheal muco-ciliary clearance of viruses by improving the ciliary beat frequency; increases levels of growth factors that play a role in the immune suppression of viruses; preserves pulmonary tissue barriers by strengthening the respiratory epithelium; and reduces the ability of SARS-CoV-2 to infect cells with ACE2 receptors that line the pulmonary tree from the pharynx to the lungs [121]. In the presence of SARS-CoV-2, zinc binds to the protein spike of the virus, changing its conformation and preventing the viral particle from functioning at multiple sites of the viral life cycle [119,120,122]. Zinc has been shown to enhance the cytotoxicity of RNA viruses. SARS-CoV-2 is an RNA virus.

The global prevalence of zinc deficiency [123] may be as high as 20% [121]. Zinc deficiency has been demonstrated in diabetics and with increasing age. An estimated 35–45% of adults >60 yrs of age have zinc intakes below the average requirement [121]. A total of 60% of elderly nursing home residents in the United States have decreased zinc intake levels [121]. These co-morbidities associated with low zinc levels are recognized risk factors for the severity of COVID-19 infection [124]. Zinc is not stored in the body and thus must be obtained daily through diet or supplements [121]. Considering the above, normal zinc serum blood levels of 0.66–1.00 mcg/mL or urine test 20–967 mcg/mL 24 h should be determined [125].

The RDA dose for elemental zinc is 11 mg po daily for men and 8 mg po daily for non-pregnant women. A typical 60 mg dose of zinc contains 11 mg of absorbable elemental zinc. Doses of elemental zinc of 50 mg twice daily have been used for prophylaxis and in the treatment of COVID-19 under the supervision of a physician to avoid possible copper deficiencies and malabsorption of other medications [122]. When zinc is used in conjunction with a zinc ionophore, e.g., quercetin, it opens the ionophores in the cell membrane of an affected cell, enhancing zinc uptake into the intracellular cytoplasm. Zinc then binds to the intracellular viral particle, inhibiting the completion of the replication cycle.

- **Quercetin.** Like famotidine, the herbal flavonoid quercetin stabilizes mast cell membranes, reducing the release of preformed histamines, chemical messengers that stimulate severe inflammation. Quercetin also inhibits the production of key enzymes responsible for manufacturing the potent leukotrienes required for a cytokine storm [126,127]. Quercetin, a broad-spectrum antiviral, antioxidant and anti-inflammatory, is a potential treatment for severe inflammation, which is one of the main life-threatening conditions in patients with COVID-19. As such, we recommend a prophylactic dose of 12.5 mg/kg, which for a 70 kg man is 1 gm po daily.
- **Acetylsalicylic acid.** The early use of aspirin (acetylsalicylic acid (ASA)) has triple effects in COVID-19 including inhibiting virus replication, anti-platelet aggregation, and anti-inflammatory changes [128]. Aspirin is considered an anti-lung injury medicant shown to shorten the length of hospital stay and reduce the incidence of cardiovascular complications [129]. Antiplatelet aggregation is important as SARS-CoV-2 has serious endovascular complications including stroke, pulmonary embolism, and myocardial infarction.

Treatment with low dose ASA for coronary artery disease, atrial fibrillation, or stroke prophylaxis confers COVID-19 protection as observed in retrospective studies [130,131,132]. Patients already on low dose ASA and having COVID-19 were significantly less likely to require admission to the intensive care unit or require use of a mechanical ventilator, and more likely to survive than patients not on ASA [130]. Based on studies specific to SARS-CoV-2, in both the prophylactic antiviral effects and effects in the treatment using ASA 75 mg po daily has been reported [130,131,132].

- **N-Acetylcysteine**. N-acetylcysteine (NAC) is used to treat critically ill septic patients and more recently patients with COVID-19 pneumonia [133]. NAC can be used in preventing COVID-19 in a dose dependent manner and reduces both inflammation and oxidative stress. The NAC inhibits in vitro binding of angiotensin-2 on the surface of the SARS-CoV-2 particles from binding to angiotensin-2 receptors on the surface of cells throughout the pulmonary tree [134]. The recommended dose for the prevention of COVID-2 is 1000–3000 mg po daily [133].

Early administration applies to patients with recent exposure to SARS-CoV-2, a newly COVID-19 positive test, or pending results of COVID-19 testing. Early administration of the above medications prophylactically is essential to help prevent the body’s immune system from progressing to an accelerated immune response with the release of excessive histamine and leukotrienes. Leukotrienes are inflammatory protein chemicals the body releases from macrophages and mast cells after the immune system encounters a viral pathogen. Leukotrienes cause the contraction of airway muscles, narrowing airways, and produce increased mucus production resulting in difficulty breathing, an important characteristic of progressive COVID-19. Patients should consult with their physician to discuss the use of these potential medications that may be prophylactic for SARS-CoV-2 infections. Although the doses presented herein provide guidelines for prescribing by the primary care provider, there exists a range of therapeutic doses.

It is important to realize that ultimately the entire world’s population will either become infected with SARS-CoV-2 and develop natural immunity or be vaccinated for SARS-CoV-2 and develop immunity secondary to the vaccine. Since there is no known natural immunity to SARS-CoV-2, it is unlikely that anyone will escape being infected [135]. Accordingly, the use of these prophylactic medications early may be important. In some ways, SARS-CoV-2 is like the varicella (chicken pox) virus. Nearly the entire population will contract chicken pox or will be vaccinated against chicken pox. Like chicken pox, the vast majority of citizens contracting SARS-CoV-2 will survive. Additionally, like chicken pox, most citizens will be mildly symptomatic or asymptomatic and not suffer from long-term sequalae. Like many viral infections, many patients with SARS-CoV-2 will have no symptoms at all. Considering the world’s population as a whole, most citizens will not only survive but the vast majority of individuals will be asymptomatic or only mildly symptomatic and develop natural immunity [136].

## 4. Discussion

On the path forward, using the guidelines described herein to advance the opening of nations may minimize the risk of morbidity and mortality from SARS-CoV-2 infections and maximize recovery from COVID-19. This document provides a framework for understanding the key principles necessary to move forward with a comprehensive plan to reduce morbidity and to increase survival while opening a nation. It should be agreed that some illness and deaths are inevitable whether the nation is recurrently closed down or whether the nation is systematically opened with precautions employed as described above. It should be understood that even with the above measures in place, a certain amount of illness and deaths will occur.

Closure has resulted in the delay or avoidance of countless primary care evaluations, diagnoses and the treatment of non-COVID-19 signs and symptoms. Health maintenance including missed cancer evaluations, diagnosis, and treatment is highly correlated with the SARS-CoV-2 pandemic. This includes missed vaccinations [137], missed glaucoma and diabetic retinopathy evaluations, diagnoses, and treatments [138]; delayed surgery [139]; progressive depression and anxiety disorders [140,141]; and suicide [142]. The magnitude of the psychiatric and mental health complications cannot be overlooked [143,144,145]. As a consequence of the above, closures have resulted in an enormous amount of morbidity and mortality [135]. Historically, there is no other viral infection that has closed nations.

Although these guidelines cover a considerable number of principles, limitations of the guidelines emerge when considering local, regional, and national variables. An example of this is the mutations of SARS CoV-2 requiring adaptation accordingly. Forward movement and learning to live with this pandemic are necessary, as was the case with epidemics and pandemics in the past [146]. The guidelines contained herein lay out a strategy for a path forward. These guidelines, when implemented, are expected to successfully move us through this pandemic and can also serve as a foundation for future pandemics.

## 5. Conclusions

These guidelines provide a primer for the types of public health interventions that should be initiated everywhere to slow the spread of the virus. The implementation of these guidelines may require consultation to address unique circumstances such as in the case of outbreaks and superspreader events that may require modification of the guidelines to meet the specific needs encountered. Taken individually, each of these guidelines educates the physician on a role they can take for creating a safe environment. Physicians collectively applying these guidelines would be expected to result in improved control of this SARS-CoV-2 pandemic, avoiding closuring and reopening a nation.

## Data Availability

None available.
